# Testing spatial working memory in pigs using an automated T-maze

**DOI:** 10.1093/oons/kvad010

**Published:** 2023-10-04

**Authors:** L M Allen, D A Murphy, V Roldan, M N Moussa, A Draper, A Delgado, M Aguiar, M A Capote, T J J Jarome, K Lee, A T Mattfeld, R Prather, T A Allen

**Affiliations:** Cognitive Neuroscience Program, Department of Psychology, Florida International University, Miami, FL, 33199, USA; Cognitive Neuroscience Program, Department of Psychology, Florida International University, Miami, FL, 33199, USA; Cognitive Neuroscience Program, Department of Psychology, Florida International University, Miami, FL, 33199, USA; Cognitive Neuroscience Program, Department of Psychology, Florida International University, Miami, FL, 33199, USA; Cognitive Neuroscience Program, Department of Psychology, Florida International University, Miami, FL, 33199, USA; Cognitive Neuroscience Program, Department of Psychology, Florida International University, Miami, FL, 33199, USA; Cognitive Neuroscience Program, Department of Psychology, Florida International University, Miami, FL, 33199, USA; Cognitive Neuroscience Program, Department of Psychology, Florida International University, Miami, FL, 33199, USA; School of Neuroscience, Virginia Polytechnic Institute and State University, Blacksburg, VA 24061, USA; School of Animal Science, Virginia Polytechnic Institute and State University, Blacksburg, VA 24061, USA; Division of Animal Sciences, College of Agriculture, Food and Natural Resources, University of Missouri, Columbia, MO 65211, USA; National Swine Resource and Research Center, University of Missouri, Columbia, MO 65211, USA; Cognitive Neuroscience Program, Department of Psychology, Florida International University, Miami, FL, 33199, USA; Division of Animal Sciences, College of Agriculture, Food and Natural Resources, University of Missouri, Columbia, MO 65211, USA; National Swine Resource and Research Center, University of Missouri, Columbia, MO 65211, USA; Cognitive Neuroscience Program, Department of Psychology, Florida International University, Miami, FL, 33199, USA; Department of Environmental Health Sciences, Robert Stempel College of Public Health, Florida International University, Miami, FL 33199, USA

**Keywords:** spatial memory, navigation, translational neuroscience, working memory, declarative memory

## Abstract

Pigs are an important large animal model for translational clinical research but underutilized in behavioral neuroscience. This is due, in part, to a lack of rigorous neurocognitive assessments for pigs. Here, we developed a new automated T-maze for pigs that takes advantage of their natural tendency to alternate. The T-maze has obvious cross-species value having served as a foundation for cognitive theories across species. The maze (17′ × 13′) was constructed typically and automated with flanking corridors, guillotine doors, cameras, and reward dispensers. We ran nine pigs in (1) a simple alternation task and (2) a delayed spatial alternation task. Our assessment focused on the delayed spatial alternation task which forced pigs to wait for random delays (5, 60, 120, and 240 s) and burdened spatial working memory. We also looked at self-paced trial latencies, error types, and coordinate-based video tracking. We found pigs naturally alternated but performance declined steeply across delays (R^2^ = 0.84). Self-paced delays had no effect on performance suggestive of an active interference model of working memory. Positional and head direction data could differentiate subsequent turns on short but not long delays. Performance levels were stable over weeks in diverse strains and sexes, and thus provide a benchmark for future neurocognitive assessments in pigs.

## INTRODUCTION

Pigs (*sus scrofa*) are frequently used in translational clinical research and have been critical to the development of many medical treatments [1, 7, 28, 51, 56], but pigs are not yet widely used in behavioral neuroscience. Part of the problem is there is a paucity of laboratory-based behavioral assessments designed for pigs. Pigs provide behavioral neuroscientists with a large animal model in which clinically relevant drug dosages and human-sized biomedical devices can be tested. For behavioral neuroscience, this affords a profound opportunity to develop new diagnostic and therapeutic tools to help with neurological disease and psychiatric disorders [2].

There are several advantages for using pigs as a model for brain-behavior relationships. For example, pigs have a large gyrencephalic brain with an overall volume that is ~1/10^th^ the size of a human brain (compared to the more commonly used mouse and rat models that have brains ~1/500^th^ and 1/400^th^ the size of a human brain, respectively; [46]). Recently, Ulyanova and colleagues [54] conducted pioneering experiments characterizing the electrophysiological properties of the pig hippocampus, which presents with similar electrophysiological signatures and laminar properties as rodents and primates [53]. They further demonstrated the value of pigs toward optimizing electrode configurations for depth recordings in large freely-behaving animals [54]. In general, pigs are anatomically, physiologically and genetically similar to humans [7]. This is important in considering models for translational behavioral neuroscience because brain–body interactions are increasingly recognized as critical variables, such as with the gut-brain axis [9, 10]. As such, it is notable that human and pig diets can be exactly matched, which is not the case for other commonly used species in behavioral neuroscience. From a research sustainability standpoint, pigs also pose fewer ethical issues than non-human primates, and laboratory resources are more common for biomedical research in pigs than other large animals at most medical research institutions. Importantly, completion of the porcine genome project in 2012 allowed for genetic manipulations in pigs to model diseases such as cystic fibrosis, heart arrhythmias, and others [21, 43]. Along these lines, our group is currently developing and validating genetic models of Alzheimer disease, strongly motivating our need for standardized behavioral assessments to maximize their potential benefits for mental health research (NIH grant AG079292).

Pigs have been demonstrated to be a robust and flexible model for learning and memory. For example, pigs have been used in a host of memory tasks that have primarily focused on spatial variables such as the spatial hole board discrimination [5], but have also been studied in non-spatial tasks such as delayed match or non-match to sample and spontaneous object recognition [29]. Mendl *et al*. [35] were the among the first groups to rigorously assess pigs in spatial memory paradigms, demonstrating that they use a systematic approach to foraging in an arena with multiple baited areas, and avoid revisiting areas previously explored. They also demonstrated that pigs can learn a simple left/right spatial discrimination in a T-maze. Pigs further demonstrate an ability to remember the location of food rewards in a win-shift task in an eight-arm radial maze set up [30]. Additionally, a manual T-maze-like apparatus was used to assess spatial delayed non-match to sample in 12- to 14-month-old pigs, who performed significantly above chance at delays of up to 300 s [38]; much longer than delays used in most rodent studies (e.g. [26, 47]).

Much of what we know about learning and memory comes from some variation of a T-maze paradigm. Typically, the maze is a T-shaped apparatus in which animals start at the base of the T and travel up the stem to the T-intersection, where they are faced with a simple forced choice: go right or left. As reviewed by d’Isa *et al*. [12], the T-maze was originally used by Robert Yerkes in 1910 to study cognition in earthworms and was later popularized by Edward Tolman for use in rodents. Tolman described an inherent tendency of animals to alternate between left and right stems on contiguous trials. This behavior is thought to be related to naturalistic foraging behavior and thus the task is considered ethologically relevant. The T-maze task informed Tolman’s conceptualization of the cognitive map in 1948 and later contributed to the theory of the hippocampus as a cognitive map [40]. The T-maze has been used in a variety of species including rats [14, 17], mice (Pioli *et al*., 2013), golden hamsters [11], cats [49], dogs [19], marmoset and macaque monkeys [18], sheep and goats [25], cows [20], rabbits, gerbils, ferrets, opossums, marmosets [12], and non-mammals including shore crabs [13], zebrafish [37], pill bugs, fruit flies, and goldfish. As a starting place for developing laboratory neurocognitive tasks for the pig, the T-Maze maximizes the comparative value for behavioral neuroscience applications because it has been used for nearly a century to assess learning and memory in rodents, despite its seemingly simple design.

Here, we describe a T-maze apparatus for pigs that can be run fully automatically and provides a rich data set for neurocognitive assessment, taking advantage of naturalistic foraging tendencies. The task parameters were designed to tax and validate spatial working memory capacities, which are critical components of both spatial navigation and episodic memory across species [3, 22]. We expected that all pigs, regardless of strain or sex, could learn the task quickly in the simple alternation phase, reflective of natural alternation behavior. We also expected pigs to perform alternation well above chance following delays, but with a significant decay in performance as delay durations increased, indicative of a working memory strategy. In addition, we hypothesized that pigs would indicate their choice earlier in the stem as training progressed, by adjusting their trajectories prior to the choice point, suggesting decisions occur earlier. As such, we utilized a sophisticated real-time overhead tracking system to surveil the position of each pig throughout each session to analyze their paths according to subsequent performance conditions. We found that pigs were asymptotic in the simple alternation task immediately, and successfully alternated following all delays, producing a memory decay function that supports our hypothesis that the task successfully probes working memory processes. However, we did not find evidence that pigs made their choices earlier than the choice point over training sessions. We report several other behavioral outcomes that help validate the automated T-maze as a rigorous neurocognitive assessment of spatial working memory in pigs. We also discuss the importance of this foundational work in future pig model studies focused on neurological and psychiatric disease and for behavioral neuroscience.

## RESULTS

### Simple alternation training

**Figure 1:**
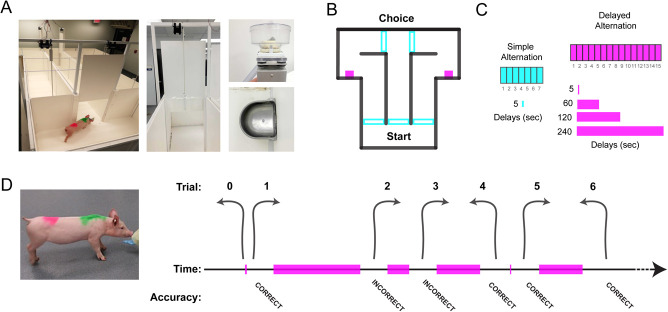
Spatial working memory task adapted for pigs using a fully automated T-maze apparatus. A large T-maze was built from PVC sheeting in the Porcine Neuroscience Facility (PNF) at Florida International University (FIU) to test working memory in pigs. (**A**) A photo of the pig T-maze (angled bird’s eye view, left panel) including one of our young Yorkshire pigs (40–50 lbs.) in the start area about to enter an open guillotine door to the stem. Close-up shots are provided of the guillotine doors (middle), pellet dispenser, and food bowel (right panels). The green and pink markings on the back are spray painted in order to track subjects via cameras and control the maze in a fully automated manner. (**B**) A simplified diagram of the T-maze showing the location of the corridors, guillotine doors (light blue rectangles), and right and left reward areas (pink boxes). (**C**) First, a simple alternation task (light blue) was used in which pigs alternated nearly continuously with the exception that the doors cycle open and closed in the start area (5 s delay), until delays (hot pink) were introduced up to 240 s between trials. (**D**) A close-up picture of a pig next to a task descriptive timeline of part of a delayed alternation session. Alternating responses was scored as correct (providing a food reward in a food bowl). Incorrect turns were unrewarded. After every choice the pig had to walk back to the start area before the next trial

In the first phase, pigs (n = 9) were trained on a fully automated simple alternation task, to familiarize themselves with the maze and rules (Fig. 1). Training occurred over six to eight sessions that were run serially across days and took advantage of the natural tendency of pigs to alternate during foraging and exploratory behaviors [30]. The tasks run automatically using guillotine doors controlled by custom-written MATLAB scripts. Unlike rodents, pigs do not require food restriction to motivate them to perform tasks for reinforcements. Briefly, sessions began with an initial sample trial (T_0_) where a food pellet reward was provided regardless of their choice at the T-junction, and set the condition for accuracy on the subsequent trial. After returning to the start area, pigs initiated the next trial (T_1_-T_n_) any time after the stem door opened. The shortest time in the start area for pigs was 5 s, as this was the minimum time required to cycle the doors. For T_1_ through T_n_, rewards were provided for accurate choices. Sessions were ended at 50 trials, lasting approximately 1 hr, or earlier if the pig demonstrated a clear lack of engagement in the task.

### Pigs were accurate from the very first session

**Figure 2:**
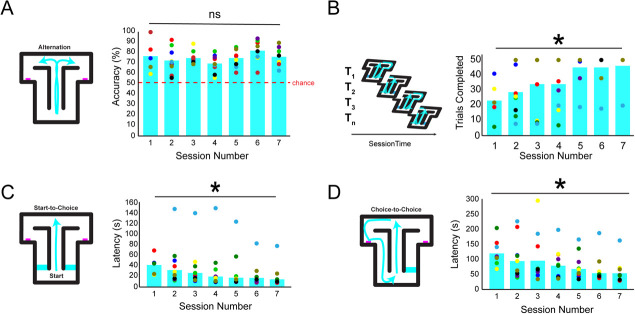
Pigs naturally alternate in the T-maze and acclimate in about a week. (**A**) Performance plotted for each pig (unique colored dot) showing all pigs were above chance on the first session and all subsequent sessions. (**B**) The number of trials each pig completed increased over sessions suggesting pigs were becoming more familiar with the apparatus. (**C**) The start-to-choice latencies decreased over sessions showing pigs were making faster choices over sessions. Note, the pale blue dot was the oldest pig at 6.5 years and was an outlier in the time he took to make choices. However, this did not evidently affect his accuracy. (**D**) Choice-to-choice latencies decreased across sessions

Overall, pigs successfully performed the simple alternation task at high levels of accuracy (75.81 ± 1.32%, n = 62 sessions; one-sample t-test against chance = 50%: t_(58)_ = 19.16, p = 4.56 × 10^−27^) demonstrating their use of alternation behaviors in the task. Female pigs had slightly higher mean performances (77.89 ± 2.22%, n = 6) than male pigs (71.99 ± 2.07%, n = 3), but this was not significantly different (t_(7)_ = 1.70, p = 0.13). Experiments were not designed to test for sex differences, but included diverse subject variables as a matter of practice. Next, accuracy was examined across sessions to evaluate learning. Figure 2A shows performance levels over the first seven sessions (S) S1–S7. Successful alternation performance in each session was determined by using one-sample t-tests versus chance (50%). Pigs performed better than chance (50%) in all sessions (S) S1–S7 (S1: t_(4)_ = 3.88, p = 0.0089; S2: t_(8)_ = 3.72, p = 2.54 × 10^−4^; S3: t_(8)_ = 11.78, p = 1.23 × 10^−6^; S4: t_(8)_ = 7.11, p = 5.05 × 10^−5^; S5: t_(8)_ = 9.03, p = 9.06 × 10^−6^; S6: t_(8)_ = 9.22, p = 7.78 × 10^−6^; S7: t_(7)_ = 8.24, p = 3.77 × 10^−5^). However, accuracy did not significantly change across sessions (S1:7: F_(6,51)_ = 1.53, p = 0.19), suggestive of the pigs’ natural tendency to alternate in the T-maze.

### Pigs completed more trials and sped up choices over sessions

Pigs acclimated their behavior over sessions evidenced by the number of trials completed per session (Fig. 2B). Specifically, a one-way ANOVA showed a significant increase in the number of trials completed across sessions (S1:S7: 14.3, 28.3, 35.6, 33.4, 44.1, 44.1 and 45.3, F_(6,51)_ = 2.64, p = 0.026). However, there was no reliable correlation between the number of trials completed and overall accuracy (Pearson’s r = 0.16, p = 0.27). Thus, the increase in trials was not related to success rates.

**Figure 3:**
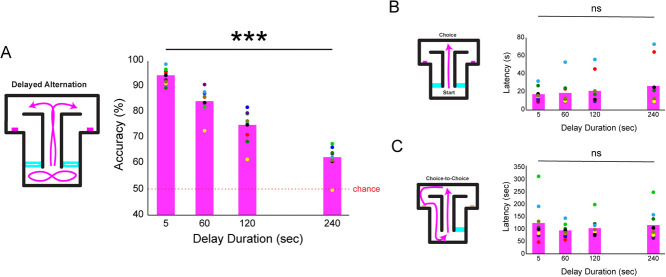
Imposing delays in the T-maze between trials parametrically taxes spatial working memory in pigs. (**A**) Performance declined as a result of increased delay times (linear regression with delay duration predicting performance: R^2^ = 0.84, p = 1.078 × 10^−17^). This demonstrates the main prediction of working memory in the delayed alternation task. (**B**) The start-to-choice latencies did not change as a function of delay duration. (**C**) The choice-to-choice latencies did not change as a function of delay duration

An analysis of the average latency to make a choice after the beginning of each trial was performed (controlling for differences in the number of trials completed; Fig. 2C). A significant decrease in the latency to make a choice relative to the start of the trial across sessions was detected by using a one-way ANOVA (S1:S7: 40.72, 29.39, 23.14, 19.56, 17.21, 13.90 and 11.64 s; F_(6,53)_ = 3.21, p = 0.011). These results demonstrate pigs completed trials faster across sessions, reflecting increased familiarity with the general task rules and apparatus. No correlation was observed between the latency to make a choice and overall accuracy (Pearson’s r = −0.0089, p = 0.95). Thus, self-paced start-to-choice latencies do not increase difficulty and/or tax working memory. Note that one pig (~6.5 years old) had to be excluded from the start-to-choice latency analysis because he was consistently much slower across sessions and was a statistical outlier, see Fig. 2C (light blue dots).

### No evidence that self-paced delays relate to performance

Lastly, we analyzed the time it took pigs to make a choice (T_n_) relative their last choice (T_n − 1_), aka choice-to-choice latencies (Fig. 2D). Conceptually, this represents the maximal possible working memory delay in the spatial alternation task, albeit entirely self-paced or spontaneous, and asks the question whether working memory was naturally burdened in the simple alternation phase. There was a significant decrease in the latency to make a choice, relative to the last choice, across sessions as determined by using a one-way ANOVA (S1:S7: 120.83, 92.10, 98.21, 77.95, 69.96, 53.39 and 49.40 s; F_(6,47)_ = 2.12, p = 0.011). To assess the relationship between choice-to-choice latencies and performance, a correlation was performed, however, no association was observed between self-paced choice-to-choice latencies and accuracy (r = 0.20, p = 0.14). These results show pigs were making faster choices, relative to their last choice, and perhaps reflecting elevated engagement in the task relative to past sessions. However, because their performance was not related to these latencies, we conclude that self-paced delays did not adversely affect working memory.

### Delayed spatial alternation testing

In the second phase, delays were imposed by restricting pigs to the start area from 5 to 240 s (Fig. 3). Delays were utilized to explicitly and parametrically burden spatial working memory. Analyses were restricted to sessions in which delays were randomly presented at 5, 60, 120 and 240 s (n = 9 pigs, 93 sessions) and do not include sessions from the first simple alternation phase. Delays could not be anticipated and were equally likely to occur on any given trial (sampling with replacement). In delay sessions, pigs completed an average of 31.3 ± 1.2 trials but this was not related to session number (r = −0.033, p = 0.75). While fewer trials per session were performed compared to the simple alternation phase, it’s worth noting that the introduction of delays increased overall session times and pigs appeared to lose interest after about 90 mins.

Most importantly, pigs performed the delayed alternation task at high overall accuracy rates (80.95 ± 0.89, n = 93 sessions, t_(92)_ = 34.87, p = 4.01 × 10^−55^). Female pigs performed similarly (79.90 ± 2.02%) to male pigs (79.66 ± 0.96%, t_(7)_ = 0.081, p = 0.94) in this task.

**Figure 4:**
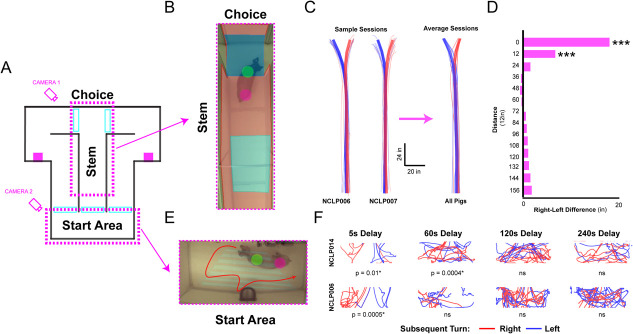
Marker-based video tracking rules out many positional confounds for memory. (**A**) A simplified diagram is shown depicting the areas analyzed to examine if coordinate tracking could reveal any path or location differences in the behavior of the pig that related to subsequent turns, performance and/or delay durations. (**B**) A picture of one of our Sinclair pigs in the stem and choice area if the maze during a choice. The green and pink circles are superimposed on the images indicating the center of mass of the color spray painted on the dorsal surface of the pig. The blue and pink shading represents tracking zones superimposed on the image for detecting when a pig is in a particular functional location (such as the choice point) to help MATLAB control the guillotine doors at the appropriate time. (**C**) Two representative pig tracking sessions are shown colored by right and left turns. The thick blue and red lines are the average path for that session and the thin lines are individual trials. The rightmost plot includes all pigs (n = 9) on right and left turn. The thick lines are the average across all pigs, and the thin lines are the average line for individual pigs. (**D**) A plot of the average difference between right and left turns for each pig. Aggregate right and left trajectories were distinguishable in the choice area, but this was not true in any other location on the stem or when separated by performance or delay duration (one-sample t-tests, df = 8, H0 = 0, ^***^, p < 0.0001). (**E**) A picture of one of our pigs from Mizzou in the start area indicating the tracking locations on the pig and tracking zones as in the stem and choice area. (**F**) Two representative pig tracking sessions showing subsequent right and left turns colored red and blue. Subsequent turns could be distinguished in the short delays indicating more of a continuous alternation trajectory, but not in the longer delay durations indicating the location of the pig in the start area is not helping with subsequent decisions

### Spatial working memory performance declines as delay durations increase

Accuracy steadily decreased across delay durations (D5 = 95.58%, D60 = 86.34%, D120 = 77.13%, and D240 = 64.12%; F_(3,371)_ = 58.30, p = 7.39 × 10^−31^; n = 93 sessions). When controlling for within-pig variability by averaging delay performances in individual pigs (n = 9), we observed the same pattern of results (D5: 95.29%, D60 = 85.77%, D120 = 75.74%, and D240 = 63.26%; F_(3,35)_ = 66.69, p = 7.36 × 10^−14^; see Fig. 3A). One-sample t-tests were used to examine performance at specific delays against chance (50%). Pigs were significantly better than chance in all delays tested (D5: t_(8)_ = 46.17, p = 2.67 × 10–11; D60: t_(8)_ = 21.06, p = 1.36 × 10^−8^; D120: t_(8)_ = 12.14. p = 9.81 × 10^−7^; D240: t_(8)_ = 7.80, p = 2.88 × 10^−5^) supporting the notion that pigs have the ability to use spatial working memory up to at least 4 min.

### Errors following correct trials (spontaneous) vs. errors following errors (perseverative)

We examined the distribution of error types spontaneous vs. perseverative errors. By definition, spontaneous errors occur after a correct trial, while perseverative errors occur after an error trial (meaning the pig turned the same way at least three times in a row). Results of this analysis showed that only 12.15 ± 1.42% of errors were perseverative. The spontaneous error count exceeded the perseverative error count in all 93 sessions. Additionally, 64 of 93 sessions (68.82%) had no perseverative errors at all. Further, perseverative errors occurred at similar rates across delays (D5: 10.71%, D60: 16.89%, D120: 10.22% and D240: 16.71%, F_(3,240)_ = 1.17, p = 0.322). Thus, delay durations did not affect task strategies. However, there were individual differences in the average percent perseverative errors across pigs as follows: 3, 8, 13, 15, 15, 11, 12, 16 and 23%. Generally, the error analysis is consistent with the idea that pigs were primarily making spontaneous errors rather than engaging in perseverative behaviors, and therefore errors were likely memory related.

### Individual differences in the time it takes to make a choice across delay durations

An analysis of the average time to complete each trial (start-to-choice latency) was performed to see if pigs took longer to make choices depending on the delay duration. During delay sessions, the average start-to-choice latency was 18.82 ± 1.41 s. Across all sessions there was a significant increase in the mean start-to-choice latency (D5 = 15.51, D60 = 16.75, D120 = 20.08 and D240 = 24.63 s; F_(3,317)_ = 3.76, p = 0.011). However, unlike most measures in the task, these start-to-choice patterns were highly varied across individual pigs (Fig. 4B) and not significant across delays when collapsed by pig (F_(3,35)_ = 0.5, p = 0.68). In fact, medians were much lower than means in the longer delay trials (D5 = 16.14, D60 = 12.85, D120 = 12.56, and D240 = 13.02 s) suggesting that some pigs skew positive on this measure, introducing variability. Specifically, start-to-choice latencies were skewed by two individual pigs that increased their time to make a choice as delay times increased (Fig. 3B, pale blue and red dots). However, the other seven pigs did not show this pattern. Overall, the patterns on the start-to-choice latencies support the idea that individual differences have a large influence over the time a pig takes to make a choice following different delay intervals.

### No evidence that delays introduced by self-paced behaviors impact performance

We next examined the self-paced time pigs took to make a choice (T_n_) relative their last choice (T_n − 1_) across delays (Fig. 3C). Across sessions (D5 = 103.96, D60 = 86.46, D120 = 93.01 and D240 = 102.22 s; F_(3,317)_ = 1.62, p = 0.18) and pigs (F_(3,35)_ = 0.59, p = 0.63) there was no significant change in the added choice-to-choice time across delays. Additionally, there was no significant correlation between self-paced added time and performance (r = 0.098, p = 0.57). Altogether, pigs paced themselves similarly at different delays, and the self-paced added time did not increase task difficulty.

### Spatial positions do not distinguish subsequent choices, accuracy, or delay durations before the choice point

We examined the pigs’ paths to assess whether positions in the stem and choice areas or start box were related to their performance (Fig. 4) in individual pigs (n = 9) and representative sessions. Paths were extracted for each pig and trial individually, and an average path was calculated for specific behavioral conditions. The first analysis separated paths by subsequent choices (right vs. left turns, Fig. 4C). This analysis should detect trajectory differences at the choice point by rule, but might also show earlier path differences (e.g., in the stem) if decisions were made before the choice point. Raw coordinate locations were used to examine trajectories of individual pigs, however, because of idiosyncratic differences (e.g., some pigs leaned a bit left, others leaned a bit right), only difference measures were compared between pigs and not the raw coordinates. The analysis was conducted in equally spaced bins (12″) starting from the choice point and ending at the start box. An average x-coordinate position was calculated for each bin and trial. These data were compared by using independent samples t-tests (right vs. left) for each bin and pig (Bonferroni corrected to maintain a FW = 0.05). Right vs. left turns could be predicted at the choice point and up to 2 ft earlier in 9/9 pigs, significantly more than expected by chance G_(1)_ = 14.98, p = 1.047 × 10^−4^ (Yates corrected). Across bins, the number of significant t-tests per bin was as follows: Bin1 = 9/9 (100%), Bin2 = 7/9 (78%) (7/9), Bin3 = 6/9 (67%), Bin4 = 2/9 (22%), Bin5–B12 = 0/9 (0%). We conclude that behavioral choices are only overtly apparent at the choice point or just before it. There was no evidence choices could be distinguished earlier than 2 ft from the choice point. Next, the average paths for each pig and condition were combined (Fig. 4C, rightmost). Although there is location variability among pigs, it was modest for average paths. Next, a distance vector was derived for right and left paths and t-tests run to determine if the paths were different for right versus left turns. Again, the only significant differences were exactly at the choice point (Bin1: t_(8)_ = 36.32, p = 3.62 × 10^−10^; Bin2: t_(8)_ = 5.10, p = 9.31 × 10^−4^; for Bin3 through Bin12: all p’s > 0.05). Next, paths were separated by correct vs. incorrect outcomes for each pig, which yielded no significant path differences at any location along the stem (significant t-tests: Bins1–12 = 0%). Lastly, we compared bin trajectories by delays (D5-D240) by using ANOVAs, and found no significant differences across paths (significant ANOVAs Bins 1–12 = 0%). Taken together, these analyses suggest that the path taken by pigs did not distinguish their eventual choice, the success of their choice, or how long they were forced to wait during different delays.

### Spatial positions in the start area on short but not long delays are related to subsequent turns

Tracking coordinates were used to examine whether the location of the pig in the start box differed based on subsequent choices. These data were plotted before the start of the trial for each pig (Fig. 4D and E) separated by delay (window size: D5, −5 to 0 s; D60-D240, −60 to 0 s). A visual examination of the plots shows that the pig locations in the start box was similar for right and left turns, but hinted at differences (especially for D5 trials in which pigs entered the start area from opposite sides during this time window). The average x-coordinate in the start area was calculated for each trial and separated by the subsequent decision (right vs. left turn). This approach tests whether pigs tended to be on the right or left side of the start area during the delay. Notably, individual pigs had idiosyncratic preferred start area locations thus inferential analyses were done individually. Independent samples t-tests were first conducted comparing right and left turns across all trials, and then for each delay independently (Bonferroni corrected for multiple comparisons, FW = 0.05). When comparing all trials, there was a significant difference in the average x-coordinate in the start area in 8/9 pigs, G_(1)_ = 15.033, p = 1.056 × 10^−4^, but this was heavily biased toward shorter delay trials (D5 = 75%, D60 = 50%, D120 = 14%, and D240 = 0% of pigs). This shows that at short delays, there was some tendency toward bias position in the start area, but this tendency diminishes steeply as time goes on and detectability was completely lost by D240.

### Head directions in the start area on short but not long delays are related to subsequent turns

**Figure 5:**
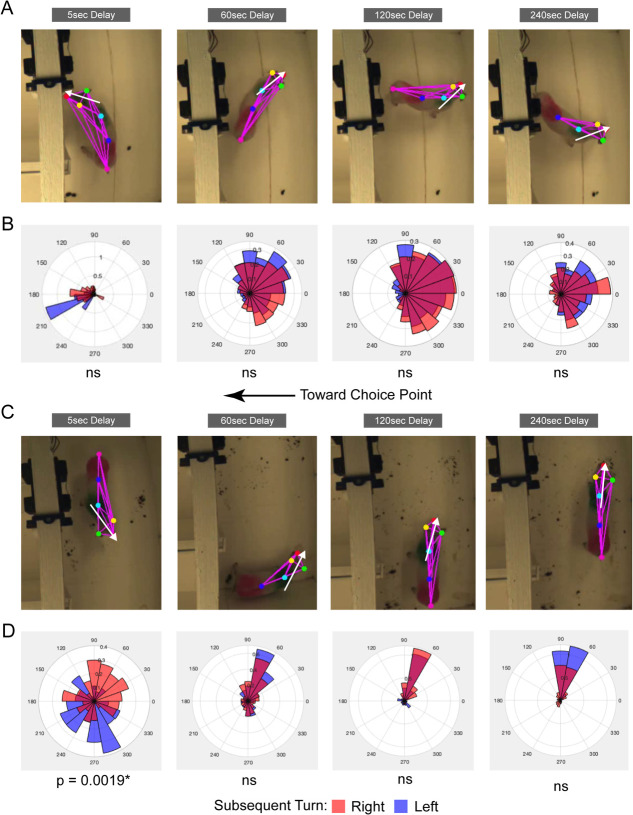
DeepLabCut-based analysis rules out many head direction confounds for a memory interpretation. Six-point tracking skeletons were acquired using DeepLabCut (red dot = nose, green dot = right ear, yellow dot = left ear, cyan dot = shoulder center, blue = hip center, hot pink = tail) for all frames. White arrows indicated the head direction calculated for that frame. In all plots and images, 0° is toward the back wall of the start area, 90° is toward the right side of the maze, 180° is toward the choice point, and 270° is toward the left side of the maze. (**A**) Sample six-point tracking skeleton screenshots for NCLP006 across delays (D5, D6, D120 and D240). B) Polar plots for all head directions calculated in corresponding delays (D5–D240). NCLP006 regularly looked toward the center stem door leading to the choice point during the first 5 s of the delays, but in all longer delays head directions tended to be toward back-wall half of the start area. Head directions for NCLP006 never differentiated subsequent right and left turns (all Watson-Williams tests, p’s > 0.05). (**C**) Sample six-point tracking skeleton screenshots for NCLP010 across delays (D5, D6, D120 and D240). (**D**) Polar plots for all head directions calculated in corresponding delays (D5–D240). NCLP010 regularly looked toward the right side before making right turns on 5 s delay trials, and the left side before making left turns on 5 s delays (Watson-Williams test, p < 0.05). However, in all longer delays head directions tended to be in the back right corner of the start area and never differentiated subsequent right and left turns (all Watson-Williams tests, p’s > 0.05)

Head directions may provide additional information in the start area about subsequent turns. A visual examination suggested that pigs sometimes looked at the center door toward the choice area and could be using this behavior to assist subsequent choices. To test this further, additional tracking data was generated in a subset of pigs (n = 6) using DeepLabCut [34] providing a six-point skeleton including the nose, right and left ears, shoulder centered, hip centered, and tail (See Fig. 5A and C). Head direction was calculated as the nose-ward direction of the perpendicular bisection of the right and left ears. Polar plots were then created with probability density histograms (Fig. 5B and D). Notably, vectors varied widely around plots and idiosyncratic tendencies were observed. Head directions were analyzed using the circ_stat toolbox in MATLAB [8]. A Watson-Williams multi-sample test for equal circular means (circ_wwtest) was used to compare head directions on right and left subsequent trials, Bonferroni corrected for multiple comparisons. There were significant differences (p’s < 0.05) in head directions in the first 5 s of the random delays in 3/6 pigs (for example see Fig. 5, first polar plot), however there were no significant differences in head direction in any of the longer delays (D60, D120 and D240; all p’s > 0.05). Thus, these results are similar to the positional analysis as there was some ability to detect differences in head direction on right versus left subsequent turns at short delays but this head direction bias was lost in longer delays.

## DISCUSSION

### Summary of main results

This paper demonstrates a new laboratory-based T-maze task for pigs that takes advantage of their natural tendency to alternate in a T-maze, reflective of basic foraging behavior, providing a rigorous test of pig memory. Our T-maze is appropriate for repeated testing of spatial working memory in pigs in a controlled laboratory environment and is fully automated to remove any experimenter bias from influencing pig behavior. We then validated this task using multiple detailed behavioral measures and found several interesting results. First, pigs alternated their turns naturally and were at near asymptotic performance the very first time we put them in the maze. This outcome is indicative of the natural tendency for pigs to forage in novel places or places where food was retrieved the longest time ago providing the most opportunity that the food replenished. Second, we found no evidence that pigs learned to alternate. Third, we found that pigs completed more trials and decreased choice latencies over their first seven session, suggesting pigs acclimated quickly to the apparatus and task. Anecdotally, pigs seemed to reduce extraneous searching behaviors during these sessions (e.g. looking for a way out of the maze or looking for food in locations other than in the reward bowls). Fourth, introducing delays between trials decreased performance. In fact, one of the main tenants of a working memory process is that performance is negatively related with time. Theoretically, this can be caused by a passive temporal decay function or by active interference from other thoughts or representations (internally or externally sourced) crowding a limited buffer capacity within working memory [6]. The fifth finding helps address these possibilities demonstrating that self-paced choice-to-choice temporal delays were not related to performance in *either* in the simple alternation task or in the delayed alternation task. This rules out passive decay models of working memory (i.e., one based solely on clock time). By contrast, imposing delays strongly affected performance despite the fact that self-paced delays were of a similar magnitude (up to a few minutes). Thus, our results favor active interference accounts of spatial working memory decline across delays in the pig T-maze, rather than one dependent on simple temporal decay [32]. In active interference models new information, originating internally and/or externally, crowds out old information from limited capacity working memory stores. Sixth, we conducted a detailed analysis of the path coordinates of the pigs in the start area, stem, and choice points. This analysis could only differentiate right and left choice when pigs were at or very near the choice point and explicitly making right or left turns. While we hypothesized *a priori* that pigs might make choices earlier than the choice point , our analyses did not support this account. Rather, it appears that the behavioral choices of the pigs are principally made at the choice point (as intended by the original T-maze designers). Seventh, we found that the location of the pig in the start area during delays was predictive of subsequent behaviors only after short delays. Importantly, short delays do not provide time for the pig to settle into their preferred waiting location and the analysis is contaminated by the contiguous return paths from the last trial. In fact, the ability to discriminate subsequent turns on short delays provides a strong validation for our analytical approaches. That is, because pigs were highly successful, this performance biases return arm positions to the opposite side relative to their next successful choice. On short delays, the predictive value of positions and head directions is likely a consequence of this starting location bias, although it might also help the pig make the next choice on these short trials. However, this behavioral bias is lost quickly as delays elapse and the initial advantage does not persist into longer delays. Thus, at short delays the ability to predict upcoming turns might simply be an artifact of successful performance coupled to our maze design.

There are many shortcomings of the present tracking-based analysis worth noting. In particular, a central tendency-based positional and head direction analysis lacks detailed pose identifications, dynamical maneuver identifications, and a fine temporal resolution. This is likely critical to assessing brief behaviors that are indicative of cognition, such as a quick glance to the target. Thus, approaches with more detailed tracking and multiple feature-based interaction terms could benefit future investigations addressing whether pigs (or any subject) demonstrate overt behaviors that can reveal upcoming decisions or other thoughts. Importantly, this is a burgeoning and promising area of investigation in the behavioral sciences [34].

### Neurocognitive assessments in pig models of neurological disease and mental health disorders

A major goal of the current work is to provide rigorous behavioral foundations and protocols for neurocognitive assessments in pig models (genetic, surgical, environmental, etc.). For decades pigs have proved invaluable as models for neurological disorders, especially traumatic brain injury, neurotoxicity, and neurovascular disorders [50, 51]. Additionally, pigs are assuming a new role as an improved animal model for understanding neurodevelopment [36], human cognition [29] and brain neurophysiology [53]. The T-maze we developed here is suitable for repeated longitudinal testing in all these models, provides several assessments of natural and learned behaviors, and provides a stable measure of spatial working memory capacities thought to be critical to declarative processes in humans [3, 22]. Importantly, our group is currently validating multiple pig models of Alzheimer disease using this T-maze task, which would not be possible without developing reliable laboratory tests of pig memory. In fact, behavioral validations in any pig model of a neurological disease will be necessary for meaningfully characterizing the pathologies and treatments that relate to the phenotypic symptoms defining of human mental health diseases. Related to aging and neurodegenerative disease the T-maze can be run longitudinally for months and likely years, and we show that it is quite suitable for old pigs (ages included here from 2 months to 6.5 years). Our older pigs (>1.5 years) all showed similar performances to the younger cohort with the exception that our oldest pig (6.5 years) was slow from a latency-to-choice perspective, but he was certainly not cognitively impaired from a memory accuracy perspective.

### Using pigs for basic behavioral neuroscience discovery

The fully automated T-maze we developed here should be a springboard for using pigs to advance basic questions in behavioral neuroscience. Scientists using the T-maze have a long history of incorporating manipulations that can be exploited to test several key brain-behavior relationships. For example, both rats and humans use place and response strategies under different training protocols, and these strategies differentially require the hippocampus and striatum, respectively [41]. Additionally, delayed-alternation tasks have been used to show that the prefrontal-reuniens-hippocampal circuitry is critical for flexible memory, especially for avoiding perseverative errors [15, 47, 55]. We measured error type distributions and found about ~7/8 errors were spontaneous (occurring after an unpredictable number of correct trials), while 1/8 were perseverative (occurring after another error), thus providing a baseline for future studies that manipulate the function or ablate the agranular medial prefrontal cortex in pigs. A clear expectation is that loss of function in the medial prefrontal regions in the pig, which are also more differentiated than in rodents, will elevate perseverative error rates. The pig T-maze provides additional opportunities for electrophysiological measures to examine the cross-species relevance of place cells, grid cells, head direction cells, sharp-wave ripples, and other memory-related electrophysiological features. We have established that pigs are capable of carrying significant chronic electrode implants [4] and have good performance following imposed delays in our fully automated T-maze, allowing for the linking of neural activity to cognition in the pig brain. Along these lines, pigs provide an additional benefit for behavioral neuroscience—training on novel complex tasks that can be comparable to those used in primates. For example, we have had preliminary success using a touchscreen apparatus for pigs in the PNF [16] testing pigs on human cognitive tasks (e.g. [23]). This approach allows us to compare memory-related neural activity in well-established rodent navigational tasks such as the T-maze, and to test whether the same signals relate to memory in human neurocognitive assessments.

### Conclusions

Pigs have a growing and important role to play in the future of behavioral neuroscience and translational clinical science targeting mental health and neurological disorders. In order to make this possible, the field is in need of laboratory-based neurocognitive tasks in pigs that take advantage of their natural behaviors, and leverage the long scientific history in behavioral, psychological and neural sciences. Here, we describe and validated a new automated T-maze that rigorously tests spatial working memory and should be part of a foundation for pig-based behavioral neuroscience going forward, in addition to other tasks we have under development. It is our hope that the pig will ultimately help advance and translate many of the findings provided by rodents and other species into the clinic, and provide an important large animal for cross-species comparison with non-primates and humans.

## METHODS

### Subjects

Subjects were male and female pigs (*Sus scrofa)* of various strains to assess performance across breeds. Female domestic pigs (n = 5; mixed-breed, primarily Yorkshire and Landrace; pink skin, white hair) were sourced from a small local farm (Walliser Pork, Inc., Wimauma, FL), weighing 30–75 lbs. on arrival, ranging from 3–5 months old. Sinclair (n = 1 male; gray skin and black hair) and Minnesota Mini (n = 2 males, n = 1 female; variegated with stripes and/or spots) pig strains were bred at the National Swine Resource and Research Center (NSRRC) at the University of Missouri in Columbia. Pigs were shipped in a USDA approved and air-conditioned livestock transport with access to food and water from Columbia, MO to Miami, FL over 1–2 days. Most pigs were 2–12 months old while two of the pigs were older (1.5 years and 6.5 years, both male) for a diversity of ages in these initial assessments. All pigs were transferred in sibling or cohort pairs to the Porcine Neuroscience Facility (PNF) at Florida International University (FIU; Miami, FL). After a 7-day quarantine, pigs were individually housed in adjacent pens to ensure equal access to food (dominance issues can affect feeding in pair-housed pigs). All pairs were housed in pens within the same holding room so that they were neighboring other pigs (retaining visual, olfactory, auditory, and tactile contact). All pigs were given a variety of environmental enrichment toys and treats for general well-being and were extensively handled by researchers and the husbandry staff. Pigs were maintained on a 12 h light-dark cycle (lights on at 5:30 am), with testing during the light phase. Pigs were given nutritionally complete Mini Pig feed (Mazuri Active Adult Mini Pig Feed, St. Louis, MO) and water (1 gallon) twice daily. Pigs were weighed regularly and daily observations on health and disposition were recorded. This experiment was conducted in compliance with the FIU Institutional Animal Care and Use Committee (IACUC) and followed all USDA requirements. 

**Table 1:** Pig T-maze components

T-MAZE PARTS	PART NUMBER	S SUPPLIER
Skeleton—1 × 1" wooden scaffolding	Internet # 207059032, Model # 21073	Home Depot
1/2 in. × 4 ft. × 8 ft. White PVC Sheet Panel	Internet # 205079515; Model # 190360	Home Depot
Reward Pellet Dispensers (2)	ENV-203	Med Associates Inc.
Dustless Precision Reward pellets (banana flavored)	F0024	Bio-Serv
Stainless steel water troughs		FIU Animal Care Facility
10 Meters Gt2 Timing Belt (6 mm width) Timing Belt with Steel Core	Amazon Standard ID #: B07ZNNR238	Amazon.com
Single Eye Bolt Counterweights for guillotine doors	Amazon Standard ID #: B07MW8PMSJ	Amazon.com
**MECHANICAL COMPONENTS**	**PART NUMBER**	**SUPPLIER**
Nema 23 Bipolar Stepper Motor	Model: 23HS45-4204S	Stepperonline; OMC Corp. Unlimited
Arduino UNO Rev3 Microcontroller board	Code: A000066; Barcode: 7630049200050	Arduino
Digital I/O board (24 channels)	NI USB-6501	National Instruments
**SOFTWARE COMPONENTS**	**VERSION NUMBER**	**SUPPLIER**
DeepLabCut	Version 2.3.5	Github/DeepLabCut
CineLyzer, including 4752 × 480 pixel USB cameras	Version 4.4.1	Plexon, Inc.
MATLAB	2021b	Mathworks, Inc.

### Handling

Pigs were handled for 5–7 days after their quarantine period before behavioral training began. In handling, experimenters allowed the pigs to sniff their gloves and gowns, pet and brush the pigs, played catch with their toys, and gave sucrose reward pellets that were used during experimentation. For behavioral training, pigs were first habituated to the T-maze and allowed to explore the maze for as long as they liked. Pellets were delivered in the choice arms to encourage exploration and help them learn where to expect rewards. Once pigs acclimated to the maze (as indicated by reduced defecation and urination, and targeting the pellet dispensers), training began.

### T-maze apparatus

Training occurred in the Porcine Neuroscience Facility (PNF) at FIU, a 1800 square foot USDA-approved facility with two housing suites, two separate behavioral training areas, and a control room for experimenters so pigs can participate in our automated task independently without humans present in the testing room.

We constructed a large, automated T-maze (17′ × 13′) in an enclosed room designed for large animal models (see Table 1). The framework for the maze is constructed from painted wooden scaffolds (2″ × 4″ and 2″ × 2″) with a few stainless-steel cross beams spanning the width of the maze for extra stability as pigs have a lot of force due to their inherent mass and can get hyper and hit the walls on occasion. The floors and walls are made with large PVC plastic sheeting to provide durable and easy-to-clean surfaces. These panels are modular and can be easily slid in and out of the scaffolding to change the shape of the maze, replace damaged panels, or for deep cleaning. The shape of the maze was built around a typical T-shape with a start area, stem, choice point and right and left reward bins (Fig. 1A and B). We added return corridors around the left and right side of the maze in order to allow the pig to return back to the start area by themselves after a completed trial. Five guillotine doors, constructed out of PVC panels, were added to control task conditions. Doors were automatically controlled with independent stepper motors coupled to Arduino UNO Rev3 microcontroller boards, and counterweighted with large painted lead fishing weights to reduce tension on the gears. Three doors separated the start area from the stem and return corridors, and right and left corridors at the choice point from the reward zones. The doors raise and lower ~3.5′ under the control of the custom-written MATLAB scripts that had access to tracking data from the four cameras (CineLyzer, Plexon, Inc.). MATLAB provided triggering signals to the Arduino microcontrollers through a NI USB-6501 24 line digital I/O board (National Instruments). Given the large size of the maze, four overhead tracking cameras were needed to simultaneously provide complete coverage of the maze. Tracking was accomplished with color detection (CineLyzer, Plexon, Inc.) targeting two painted areas on the back (dorsal surface) of each pig between the shoulder blade and near the rump using livestock grade fluorescence spray paint about 10 cm in diameter. These two tracking points provided data for location and direction, and were also used to detect when pigs entered key zones including the start area, choice point and right and left reward zones in order to appropriately coordinate the opening and closing of the guillotine doors to prevent backtracking. At the start of a trial, all doors were closed and the pig was in the start area. Code for the behavioral tasks is available here: https://github.com/T2xDel/PigTMaze/.

### Training

Pigs enter the maze through a side door in the start area. In some pigs the start area was equipped with a water bowl to keep pigs hydrated but this is not a critical feature, although seemed necessary for larger older pigs. The task was motivated by food rewards, but no deprivation was necessary. Rewards were given only for correct choices, one to three reward pellets (Dustless Precision Pellets Primate, Grain based, Banana flavor, 300 mg; Bio-Serv, Flemington, NJ), and were automatically dispensed (Med Associates Pellet Dispenser 300MG W/IR) into a food bowl located in the choice arm. The number of pellets depended on each pig, but was held constant throughout the experiment for each pig. The automated T-Maze reduces many possible human errors that may occur while running a session, as well as removes the confounding variables introduced with excessive human interference such as the ‘Clever Hans’-like phenomenon. Each session was monitored through cameras and sounds by at least one researcher in a control room located off the testing room to ensure that everything was functioning properly.

Pigs completed one to eight sessions of simple alternation training before delays were introduced in later sessions. Each session of simple alternation training consisted of up to 50 trials, up to 1–2 h in the maze depending on the task, and was suspended if the pig was clearly not performing (although this rarely occurred). In the simple alternation task, the objective is to near continuously alternate between choice arms to attain the reward, with the exception that the pig had to wait for the doors to cycle closed first before opening again in the start area since this is a critical feature of the delay phase. However, unlike other spontaneous or continuous alternation tasks (e.g. [12]), this discrete trial version forces pigs to stop in a fully enclosed start area before beginning the next trial and was food-rewarded. Trials were initiated by the start door opening to the middle stem. When the pig was 1 m into the middle stem, the start door closed, and the choice doors open. Once they entered a choice arm, the choice doors were closed behind them to prevent backtracking into the middle stem or the other choice arm. On the first trial, any choice made was rewarded. To receive rewards on subsequent trials, pigs had to ‘alternate’ by entering the side of the stem opposite to their previous choice. The return door to the start box opened, and when the tracking software detected both shoulder and hip markers in the start box, all doors were closed, completing the trial. The next trial was initiated after the retention interval or delay (in this case 5 s) whereby the start door opened into the middle stem and the pig made their next choice. Once pigs reached ~75% correct performance on 3 consecutive sessions, they advanced to the first phase of delayed alternation training in which delays up to two mins were randomly introduced, holding pigs in the start area for 5 s, 60 s, 90 s, or 120 s (not all delays were always used in this phase) presented randomly with replacement. Once pigs completed three sessions with over 75% correct overall, they moved on to the second phase for delayed alternation testing. In this phase delays of 5 s, 60s, 120 s, and 240 s were imposed in the Start Area and presented randomly with replacement following recommended practices when designing temporal tasks [27]. Pigs completed 3–5 sessions in Phase 1, and 12–15 sessions in Phase 2 of the Delayed Alternation training. After each training session, pigs were returned back to their holding room and the maze was thoroughly cleaned, first with a broom and afterwards with a mop soaked in disinfectant diluted in water to eliminate excrement, dirt, and saliva from the walls and floor of the maze.

### Data analysis

Each session was recorded from beginning to end on the four overhead cameras. The same MATLAB script that ran the task recorded behavioral parameters including conditions, accuracy and latencies. Scripts were written to batch process these data using basic statistics including mean, medians, standard deviations, range, etc. Inferential tests were conducted using t-tests, ANOVAs, goodness-of-fit tests, and linear regressions with native MATLAB functions including the Statistics Toolbox and Curve Fitting Toolbox. Videos were recorded in .avi files from overhead cameras which captured pigs’ positions in the maze. Marker-based tracking coordinates were analyzed using custom MATLAB scripts, including unwarping the fish-eye images that fed into the cameras using the Computer Vision Toolbox automatically using parameters derived from multiple 3D images of a checkerboard in each of the cameras and smoother with a 2D-filter to eliminate outlier points. Plots were made in MATLAB (2021B) and Adobe Illustrator (Version 26) for visualization. DeepLabCut [34] was used to retrack the data for head direction estimates frame-by-frame. A total of 2400+ extracted video frames were manually labeled with six body markers including the nose, right and left ears, shoulder center, hip center, and tail (See Fig. 5A and C). These served as the training dataset for our tracking network to ultimately obtain cartesian coordinates of all markers per frame connected by a skeleton across time. Tracking was refined by correcting samples of outlier markers with an accuracy-of-prediction likelihood below 90%. Circular data were analyzed using the circ_stat toolbox in MATLAB [8] and plotted using polar plot histograms (MATLAB). Trials were sorted by timestamps aligned with the behavioral data tables and through re-scoring frame numbers from videos by hand.

## Funding

This work was supported by National Institutes of Aging (NIA) grant R21 AG079292 to T.A.A., K.L., and T.J.J., funding for the National Swine Resource and Research Center (NSRRC) from NIA and the Office of the Director U42 OD011140 to R.S.P. and Kevin D. Wells, and funding from the Feinberg Foundation to T.A.A. A special thanks to all members of the Allen Lab, especially Abdiel Vasallo Veliz, Courtney Afram-Gyenin, Karolina Euqueres, Josh Vargas, and Ethan Tieu who have contributed to various aspects of establishing the Porcine Neuroscience Facility (PNF) at FIU. We would also like to thank the Animal Care Facility and Dr. Horatiu Vinerean. This project followed the ARRIVE guidelines.

## AUTHOR CONTRIBUTIONS

L.M. Allen, T.A. Allen (Conceptualization), L.M. Allen, V. Roldan, A.T. Mattfeld, A. Draper, and T.A. Allen (Methodology), L.M. Allen, D.A. Murphy, M.N. Moussa, V. Roldan, A. Draper, A. Delgado, M. Aguiar, and M.A. Capote (Investigation), V. Roldan, A.T. Mattfeld, T.A. Allen, (Data Analysis), L.M. Allen, V. Roldan, A.T. Mattfeld, T.A. Allen (Writing), L.M. Allen, V. Roldan, T.J.J. Jarome, K. Lee, R. Prather, A.T. Mattfeld, T.A. Allen (Review and Editing), T.J.J. Jarome, K. Lee, R. Prather, T.A. Allen (Funding acquisition), K. Lee, R. Prather, T.A. Allen (Resources), T.A. Allen (Supervision)

## CONFLICT OF INTEREST STATEMENT

The authors declare no competing financial interests.

## Supplementary Material

Web_Material_kvad010
