# Supplementary material for: Testing spatial working memory in pigs using an automated T-maze
Source: Oxf Open Neurosci. 2023 Oct 4;2:kvad010. doi: 10.1093/oons/kvad010 (PMC10913826; doi:10.1093/oons/kvad010)
Supplement: Web_Material_kvad010 [file Web_Material_kvad010.zip › Reviewers comments - Supplementary Material - OXFNSC-2023-007.R1.docx]

**REVIEWER COMMENTS**

**OXFNSC-2023-007.R1 - Testing Spatial Working Memory in Pigs using an Automated T-Maze**

**Original submission - OXFNSC-2023-007**

**Reviewer 1**

Comments to the Author

SUMMARY:

While various cognitive tests have been performed in the pig, it is surprising that classic alternation on t-mazes have not. This study describes how pigs behave on this task as a function of delay interval and this automated version uses tracking to precisely identify the presumed choice point. In addition, this study presents a methodology and hardware/software system that other laboratories could use for investigations of pig cognition.

Major Points

While this manuscript focuses on behavior, a stated goal is to provide the research community with a method/apparatus for assessing pig cognition. It is clear that considerable effort went into the design of the apparatus and the control and post-processing software. As a result, providing more information in this regard would benefit the research community. For example, a list of parts, part numbers, and suppliers for the apparatus is essential. This could be provided as a table. In addition, computer code used for operating the t-maze should be provided as an open-access repository on github so that others in the community could adopt this paradigm and facilitate reproducibility of the results.

Animal Heading: It is great that a detailed analysis of position was performed, and this analysis indicated a location bias during the delay interval. It would be important to augment this with an analysis of heading as the x-coordinate position alone does not indicate where the pig’s head is facing. Even if positions overlap for the left-right choice, the pig’s head/body could systematically face the target direction during the delay phase. This may not be evident in the x position. Heading angle computation should be straightforward given the 2 colors used on the pig to indicate front and back. I recall heading being reported as indicating working memory performance by Jerzy Konorski with dogs with prefrontal lesions (their heading was used during the delay interval in an embodied way to solve the task). Please analyze heading during the Start delay periods (e.g., replace position with heading in Figure 4F). Circular statistics could be used to test whether heading was different for right vs. left choices could be used to determine if there was a difference (e.g., Matlab circstat toolbox by Berens).

Related to the above, the central question pertaining to Figure 4f is the question of whether there is information in the pig’s body position during Start that predicts the future choice. A more powerful way to address this that would complement the analysis above would be to include booth the x,y position and heading as independent variables into a regression with the future choice (Left/Right) as the outcome variable (perhaps logistic regression). A separate regression could be performed for each delay interval as was performed for the t-tests and analyzed as done in Figure 4f.

Figure 2b: The y axis is mislabeled. It should be start-to-choice latency, not accuracy.

Since self-paced delays had no effect on performance, the authors indicate that data supports the proactive interference model of working memory. What is the proactive interference model? Please describe this model. Only one line in the discussion and the abstract mention the model with no overall description of the model itself. “e.g., Thus, our results favor a proactive interference account of the spatial working memory decay in the present T-maze task (Lewandowsky et al., 2009)”

Figure 4d: There is no axis indicating the units for the x axis (counts of significant differences? T values? Inches? Percent of significant t-tests?) or where zero (or chance) would be given that this is stated to be the difference between left and right. Does the thick black line indicate zero? I thought that since differences within pig were computed then this could have positive or negative values but from the main text it seems that these are ether counts of the number of significant t-tests or the t values – so only positive. The specific measure should be clarified in the caption for 4d. The overall analysis is fairly clear in the main text.

Related to the above: “Across bins, the number of significant t-tests per bin was as follows: Bin1 = 100%, Bin2 = 78%, Bin3 = 67%, Bin4 = 22%, Bin5 - B12 = 0% of pigs”. The percentages are listed here, not the number of significant t-tests.

Minor Points

Figure 1: I think that Ci and Cii could be combined more compactly in a single subplot. A lot of space is taken up by these simple schematic figures.

Figure 1: It would be nice to see a more close-up view of the pulley apparatus used to pull the guillotine doors and the feeding apparatus used to feed the pigs.

Figure 4c: A scale bar would be useful (readers can try to align the image to Figure 4d, but it would be nice to have a scale bar in 4c).

Figure 4d: The “bin distance” label on the y axis is a little confusing. I think it would be clearer to label the y axis as just “Distance (inches)” and then keep the subheading that indicates that each bin was 12”.

Minor: Technically, stating that “Female pigs were slightly more accurate (77.89 ± 2.22%, n = 6) than 110 male pigs (71.99 ± 2.07%, n = 3)” is not accurate given that there was no significant difference between groups. You could state that the mean performance was higher in the females and then state that the difference was not significant.

SWR should be defined.

This heading was a little confusing: “No evidence that self-paced added delay durations impact performance”. Could be rewritten for clarity. ‘e.g., delays introduced by self-paced trials…’

An exceedingly minor point that can be ignored: Seems like ‘overkill’ that video tracking + Matlab were used to control the apparatus when simple IR sensors with an Arduino or Raspberry Pi would be a simpler and less expensive solution for maze control. The camera of course is essential for trajectory tracking, just not necessary for experiment control.

Page 7: only derivative measures were compared between pigs. Please be clear. I assume that by this it is meant that the difference (Euclidean in x) between the left and right was computed per pig and that is what was averaged across pigs?

This wording could be clarified “The durations were chosen to include multiple points double the last and followed basic behavioral guidelines for temporal behavioral tasks (Jayachandran & Allen, 2022).”

No citation for pill bugs, fruit flies, and goldfish on page 3. Bees should be added- there is a considerable literature on bee decision making using the T or the Y configured as a T (central choice arm) version.

It is not entirely clear what all of the differences between Phase 1 and Phase 2 were beyond the 90sec interval and 120sec interval in Phase 2. Was randomization with replacement used in Phase 1 or some other approach?

Page 2: Typo with mouse and rat models having brains ~1/400th and ~1/500th the size of a human brain, respectively. The order must be reversed.

Page 5: Minor but the statement, “pigs became more comfortable” verges on anthropomorphizing.

Page 9: Typo on line 8, “main tenants” should be “main tenets”

Page 9 (top): Authors start with ‘First,’ and then jump right to ‘Third,’ with no ‘Second,’. This is probably just a typo.

For a given pig, was the side bias on trial 0 consistent across sessions? Just curious as to whether pigs have a strong initial-trial side bias that carries over from day to day.

**Reviewer 2**

Comments to the Author

Authors present methods and supporting data for a new fully-automated T-maze for pigs. In addition to providing a thorough explanation of the translational value of pig models in behavioral neuroscience, authors demonstrate that performance patterns for the pigs were stable, were sensitive to delays, and were not subject to ‘non-mnemonic’ confounds using behavioral tracking methods. Collectively, I thought this was a convincing demonstration that pigs could be used as models for working memory and beyond. I include only minor points to enhance clarity.

The curious finding of behavioral tracking position/direction being predictive in the choice point but not start box (except for short delays) needs some further description. This result is also not mentioned in the Abstract). At first I thought that the pig could be adopting a strategy to point in the direction of its future choice in short delay trials, but then I realized that it couldn’t really do this because delays were not presented in blocks, but rather they were inter-mixed within a single session (Fig 1D). In explanation of this, authors write, “Thus, at short delays the ability to predict upcoming turns is an artifact of a continuous trajectory” but I do believe that the possibility of some kind of prospective choice strategy to pass the time (in this case short time) given previous trial success could be explored as a possibility. Does this tendency to predict performance based on automated tracking drop off steeply within the trial? More details can be provided. All I could find on this in the Results was “This shows that at short delays, there was some tendency toward bias position in the start area, but this tendency diminishes quickly as time goes on and is completely lost by D240.”

Authors mention in discussion they measured error type, finding that ~7/8 errors were spontaneous, while 1/8 were perseverative (occurring after another error). This is interesting. Can authors provide more detail on individual animal performance on new errors vs. perseverative errors or some kind of trial +1 performance or win-stay, lose-switch strategy?

Minor

I would replace wording like ‘became more comfortable’ with ‘became acclimated’ to describe the effect of increasing familiarity and faster choice latencies.

Incomplete sentence: last sentence before the section “Neurocognitive assessments in pig models of neurological disease and mental health disorders” in the Discussion.

Figure 2C caption, delete ‘time’ as latencies are mentioned.

Figure 4F caption, start ‘arm’?

**Decision letter - OXFNSC-2023-007**

01-Jul-2023

Dear Dr. Allen,

Manuscript ID OXFNSC-2023-007 entitled "Testing Spatial Working Memory in Pigs using an Automated T-Maze" which you submitted to the Oxford Open Neuroscience, has been reviewed. The comments of the reviewer(s) are included at the bottom of this letter.

The reviewers have recommended publication pending some minor revisions to your manuscript. Therefore, I invite you to respond to the reviewers' comments and revise your manuscript.

Please note that this journal operates with transparent peer review. This means that if your submission is accepted for publication, the full peer review history of your article will publish online alongside your article. This includes reviewer comments, editor decision letters, and your author responses.

To revise your manuscript, log into https://mc.manuscriptcentral.com/oxfnsc and enter your Author Centre, where you will find your manuscript title listed under "Manuscripts with Decisions." Under "Actions," click on "Create a Revision." Your manuscript number has been appended to denote a revision.

You may also click the below link to start the revision process (or continue the process if you have already started your revision) for your manuscript. If you use the below link you will not be required to login to ScholarOne Manuscripts.

*** PLEASE NOTE: This is a two-step process. After clicking on the link, you will be directed to a webpage to confirm. ***

https://mc.manuscriptcentral.com/oxfnsc?URL_MASK=ef890240a3684e499fd54257c0445547

You will be unable to make your revisions on the originally submitted version of the manuscript. Instead, revise your manuscript using a word processing program and save it on your computer. Please also highlight the changes to your manuscript within the document by using the track changes mode in MS Word or by using bold or colored text.

Once the revised manuscript is prepared, you can upload it and submit it through your Author Centre.

When submitting your revised manuscript, you will be able to respond to the comments made by the reviewers in the space provided. You can use this space to document any changes you make to the original manuscript. In order to expedite the processing of the revised manuscript, please be as specific as possible in your response to the reviewers.

IMPORTANT: Your original files are available to you when you upload your revised manuscript. Please delete any redundant files before completing the submission.

Because we are trying to facilitate timely publication of manuscripts submitted to the Oxford Open Neuroscience, your revised manuscript should be uploaded as soon as possible. If it is not possible for you to submit your revision in a reasonable amount of time, we may have to consider your paper as a new submission.

Once again, thank you for submitting your manuscript to the Oxford Open Neuroscience and I look forward to receiving your revision.

Sincerely,

Dr. Alicia Izquierdo

Senior Editor, Oxford Open Neuroscience

aizquie@psych.ucla.edu

Reviewer: 1

Comments to the Author

SUMMARY:

While various cognitive tests have been performed in the pig, it is surprising that classic alternation on t-mazes have not. This study describes how pigs behave on this task as a function of delay interval and this automated version uses tracking to precisely identify the presumed choice point. In addition, this study presents a methodology and hardware/software system that other laboratories could use for investigations of pig cognition.

Major Points

While this manuscript focuses on behavior, a stated goal is to provide the research community with a method/apparatus for assessing pig cognition. It is clear that considerable effort went into the design of the apparatus and the control and post-processing software. As a result, providing more information in this regard would benefit the research community. For example, a list of parts, part numbers, and suppliers for the apparatus is essential. This could be provided as a table. In addition, computer code used for operating the t-maze should be provided as an open-access repository on github so that others in the community could adopt this paradigm and facilitate reproducibility of the results.

Animal Heading: It is great that a detailed analysis of position was performed, and this analysis indicated a location bias during the delay interval. It would be important to augment this with an analysis of heading as the x-coordinate position alone does not indicate where the pig’s head is facing. Even if positions overlap for the left-right choice, the pig’s head/body could systematically face the target direction during the delay phase. This may not be evident in the x position. Heading angle computation should be straightforward given the 2 colors used on the pig to indicate front and back. I recall heading being reported as indicating working memory performance by Jerzy Konorski with dogs with prefrontal lesions (their heading was used during the delay interval in an embodied way to solve the task). Please analyze heading during the Start delay periods (e.g., replace position with heading in Figure 4F). Circular statistics could be used to test whether heading was different for right vs. left choices could be used to determine if there was a difference (e.g., Matlab circstat toolbox by Berens).

Related to the above, the central question pertaining to Figure 4f is the question of whether there is information in the pig’s body position during Start that predicts the future choice. A more powerful way to address this that would complement the analysis above would be to include booth the x,y position and heading as independent variables into a regression with the future choice (Left/Right) as the outcome variable (perhaps logistic regression). A separate regression could be performed for each delay interval as was performed for the t-tests and analyzed as done in Figure 4f.

Figure 2b: The y axis is mislabeled. It should be start-to-choice latency, not accuracy.

Since self-paced delays had no effect on performance, the authors indicate that data supports the proactive interference model of working memory. What is the proactive interference model? Please describe this model. Only one line in the discussion and the abstract mention the model with no overall description of the model itself. “e.g., Thus, our results favor a proactive interference account of the spatial working memory decay in the present T-maze task (Lewandowsky et al., 2009)”

Figure 4d: There is no axis indicating the units for the x axis (counts of significant differences? T values? Inches? Percent of significant t-tests?) or where zero (or chance) would be given that this is stated to be the difference between left and right. Does the thick black line indicate zero? I thought that since differences within pig were computed then this could have positive or negative values but from the main text it seems that these are ether counts of the number of significant t-tests or the t values – so only positive. The specific measure should be clarified in the caption for 4d. The overall analysis is fairly clear in the main text.

Related to the above: “Across bins, the number of significant t-tests per bin was as follows: Bin1 = 100%, Bin2 = 78%, Bin3 = 67%, Bin4 = 22%, Bin5 - B12 = 0% of pigs”. The percentages are listed here, not the number of significant t-tests.

Minor Points

Figure 1: I think that Ci and Cii could be combined more compactly in a single subplot. A lot of space is taken up by these simple schematic figures.

Figure 1: It would be nice to see a more close-up view of the pulley apparatus used to pull the guillotine doors and the feeding apparatus used to feed the pigs.

Figure 4c: A scale bar would be useful (readers can try to align the image to Figure 4d, but it would be nice to have a scale bar in 4c).

Figure 4d: The “bin distance” label on the y axis is a little confusing. I think it would be clearer to label the y axis as just “Distance (inches)” and then keep the subheading that indicates that each bin was 12”.

Minor: Technically, stating that “Female pigs were slightly more accurate (77.89 ± 2.22%, n = 6) than 110 male pigs (71.99 ± 2.07%, n = 3)” is not accurate given that there was no significant difference between groups. You could state that the mean performance was higher in the females and then state that the difference was not significant.

SWR should be defined.

This heading was a little confusing: “No evidence that self-paced added delay durations impact performance”. Could be rewritten for clarity. ‘e.g., delays introduced by self-paced trials…’

An exceedingly minor point that can be ignored: Seems like ‘overkill’ that video tracking + Matlab were used to control the apparatus when simple IR sensors with an Arduino or Raspberry Pi would be a simpler and less expensive solution for maze control. The camera of course is essential for trajectory tracking, just not necessary for experiment control.

Page 7: only derivative measures were compared between pigs. Please be clear. I assume that by this it is meant that the difference (Euclidean in x) between the left and right was computed per pig and that is what was averaged across pigs?

This wording could be clarified “The durations were chosen to include multiple points double the last and followed basic behavioral guidelines for temporal behavioral tasks (Jayachandran & Allen, 2022).”

No citation for pill bugs, fruit flies, and goldfish on page 3. Bees should be added- there is a considerable literature on bee decision making using the T or the Y configured as a T (central choice arm) version.

It is not entirely clear what all of the differences between Phase 1 and Phase 2 were beyond the 90sec interval and 120sec interval in Phase 2. Was randomization with replacement used in Phase 1 or some other approach?

Page 2: Typo with mouse and rat models having brains ~1/400th and ~1/500th the size of a human brain, respectively. The order must be reversed.

Page 5: Minor but the statement, “pigs became more comfortable” verges on anthropomorphizing.

Page 9: Typo on line 8, “main tenants” should be “main tenets”

Page 9 (top): Authors start with ‘First,’ and then jump right to ‘Third,’ with no ‘Second,’. This is probably just a typo.

For a given pig, was the side bias on trial 0 consistent across sessions? Just curious as to whether pigs have a strong initial-trial side bias that carries over from day to day.

Reviewer: 2

Comments to the Author

Authors present methods and supporting data for a new fully-automated T-maze for pigs. In addition to providing a thorough explanation of the translational value of pig models in behavioral neuroscience, authors demonstrate that performance patterns for the pigs were stable, were sensitive to delays, and were not subject to ‘non-mnemonic’ confounds using behavioral tracking methods. Collectively, I thought this was a convincing demonstration that pigs could be used as models for working memory and beyond. I include only minor points to enhance clarity.

The curious finding of behavioral tracking position/direction being predictive in the choice point but not start box (except for short delays) needs some further description. This result is also not mentioned in the Abstract). At first I thought that the pig could be adopting a strategy to point in the direction of its future choice in short delay trials, but then I realized that it couldn’t really do this because delays were not presented in blocks, but rather they were inter-mixed within a single session (Fig 1D). In explanation of this, authors write, “Thus, at short delays the ability to predict upcoming turns is an artifact of a continuous trajectory” but I do believe that the possibility of some kind of prospective choice strategy to pass the time (in this case short time) given previous trial success could be explored as a possibility. Does this tendency to predict performance based on automated tracking drop off steeply within the trial? More details can be provided. All I could find on this in the Results was “This shows that at short delays, there was some tendency toward bias position in the start area, but this tendency diminishes quickly as time goes on and is completely lost by D240.”

Authors mention in discussion they measured error type, finding that ~7/8 errors were spontaneous, while 1/8 were perseverative (occurring after another error). This is interesting. Can authors provide more detail on individual animal performance on new errors vs. perseverative errors or some kind of trial +1 performance or win-stay, lose-switch strategy?

Minor

I would replace wording like ‘became more comfortable’ with ‘became acclimated’ to describe the effect of increasing familiarity and faster choice latencies.

Incomplete sentence: last sentence before the section “Neurocognitive assessments in pig models of neurological disease and mental health disorders” in the Discussion.

Figure 2C caption, delete ‘time’ as latencies are mentioned.

Figure 4F caption, start ‘arm’?

Associate Editor: 1

Comments to the Author:

Overall, both reviewers expressed enthusiasm about these data, and both recommended more detail on methods. Reviewer 1 suggested additional analyses of head direction or 'heading' and Reviewer 2 suggested an additional analysis on subsequent trials/errors. These analyses could be very informative.

**Author response**

> We thank the reviewers for their time and thoughtful comments. We have addressed all Reviewers’ comments below in a point-by-point fashion (our responses our denoted by placing a “>” at the beginning of statements, and with blue text). We made corresponding changes in the revised manuscript (denoted with blue text). The updated manuscript now includes a new analysis of head direction during delays periods in the start area using DeepLabCut tracking, a Github link for the behavioral code, and a new Table with the major parts and suppliers needed to make the pig T-maze, in addition to changes to text as suggested. We believe this revised manuscript has been significantly improved.

Reviewer: 1

Major Points

While this manuscript focuses on behavior, a stated goal is to provide the research community with a method/apparatus for assessing pig cognition. It is clear that considerable effort went into the design of the apparatus and the control and post-processing software. As a result, providing more information in this regard would benefit the research community.

For example, a list of parts, part numbers, and suppliers for the apparatus is essential. This could be provided as a table.

> We added Table 1 listing the major parts for the pig T-maze.

In addition, computer code used for operating the t-maze should be provided as an open-access repository on github so that others in the community could adopt this paradigm and facilitate reproducibility of the results.

> The MATLAB scripts for the simple and delayed alternation tasks have been made available at https://github.com/T2xDel/PigTMaze/.

Animal Heading: It is great that a detailed analysis of position was performed, and this analysis indicated a location bias during the delay interval. It would be important to augment this with an analysis of heading as the x-coordinate position alone does not indicate where the pig’s head is facing. Even if positions overlap for the left-right choice, the pig’s head/body could systematically face the target direction during the delay phase. This may not be evident in the x position. Heading angle computation should be straightforward given the 2 colors used on the pig to indicate front and back. I recall heading being reported as indicating working memory performance by Jerzy Konorski with dogs with prefrontal lesions (their heading was used during the delay interval in an embodied way to solve the task). Please analyze heading during the Start delay periods (e.g., replace position with heading in Figure 4F). Circular statistics could be used to test whether heading was different for right vs. left choices could be used to determine if there was a difference (e.g., Matlab circstat toolbox by Berens).

> We agree that a positional analysis of the pigs in the start area is not exhaustive of the possible overt behavioral indicators of future turns. Generally, one of our two color markers on the pig was more reliable than the other depending on a pig’s skin color precluding the analysis using this tracking data. In order to explore head direction, we instead generated new tracking data using DeepLabCut creating six-point skeletons (nose, right and left ears, shoulder centered, hip centered, and tail). We then derived head direction using the nose-ward perpendicular bisection of the right and left ears (see new Figure 5). We aligned trial events to their frames by hand scoring for precision and analyzed whether head directions differentiated right and left subsequent turns. As with the positional analysis, this was sometimes detectable at the shortest delay (5s) but not at longer delays. We provide detailed polar plots which reinforce this finding.

> However, we do believe more sophisticated statistical tools might be useful for revealing any predictive value in head directions in working memory tasks. Along these lines, head direction may reveal subtle behaviors indicative of subsequent turns but likely represent rare events better estimated with more sophisticated pose or motor maneuver identification analysis, but believe this is beyond the scope of the present manuscript. Some short-comings of our simple positional and head direction analysis are now included in the Discussion (Lines 303-310), in addition to some future directions.

> We added the following to the Results (Lines 246-261):

> “Head directions in the start area on short but not long delays are related to subsequent turns

Head directions may provide additional information in the start area about subsequent turns. A visual examination suggested that pigs sometimes looked at the center door toward the choice area and could be using this behavior to assist subsequent choices. To test this further, additional tracking data was generated in a subset of pigs (n = 6) using DeepLabCut (Mathias et al., 2018) providing a six-point skeleton including the nose, right and left ears, shoulder centered, hip centered, and tail (See Figure 5A&C). Head direction was calculated as the nose-ward direction of the perpendicular bisection of the right and left ears. Polar plots were then created with probability density histograms (Figure 5B&D). Notably, vectors varied widely around plots and idiosyncratic tendencies were observed. Head directions were analyzed using the circ_stat toolbox in MATLAB (Behrens, 2019). A Watson-Williams multi-sample test for equal circular means (circ_wwtest) was used to compare head directions on right and left subsequent trials, Bonferroni corrected for multiple comparisons. There were significant differences (p’s < 0.05) in head directions in the first 5 sec of the random delays in 3/6 pigs (for example see Figure 5, first polar plot), however there were no significant differences in head direction in any of the longer delays (D60, D120 and D240; all p’s > 0.05). Thus, these results are similar to the positional analysis as there was some ability to detect differences in head direction on right versus left subsequent turns at short delays but this head direction bias was lost in longer delays.”

Related to the above, the central question pertaining to Figure 4f is the question of whether there is information in the pig’s body position during Start that predicts the future choice. A more powerful way to address this that would complement the analysis above would be to include booth the x,y position and heading as independent variables into a regression with the future choice (Left/Right) as the outcome variable (perhaps logistic regression). A separate regression could be performed for each delay interval as was performed for the t-tests and analyzed as done in Figure 4f.

> We agree with the reviewer that this suggestion, or a similar analysis integrating circular and linear data into a regression, could reveal the subsequent choices of our pigs. We plan to look at this in future studies as we become develop expertise in detailed pose estimations and identifications, dynamical maneuver identifications, and better rig design set ups to take advantage of this technology (such as using multiple camera angles).

Figure 2b: The y axis is mislabeled. It should be start-to-choice latency, not accuracy.

> We checked the axis labels in Figure 2B which appear to be correct, but Figure 3B was updated accordingly.

Since self-paced delays had no effect on performance, the authors indicate that data supports the proactive interference model of working memory. What is the proactive interference model? Please describe this model. Only one line in the discussion and the abstract mention the model with no overall description of the model itself. “e.g., Thus, our results favor a proactive interference account of the spatial working memory decay in the present T-maze task (Lewandowsky et al., 2009)”

> To address this issue, we have expanded and rephrased the text in the Discussion on Lines 256-266, copied below for ease of reading. The interference account of working memory suggests it is capacity limited and not simply a fading trace. We did not intend to present a formal model, but rather discuss that our results are consistent this aspect of working memory as described by Baddeley and others.

> “… one of the main tenants of a working memory process is that performance is negatively related with time. Theoretically, this can be caused by passive temporal decay or by active interference from other thoughts or representations (internally or externally sourced) crowding the limited buffer capacity in working memory (Baddeley, 2000). The fifth finding helps address these possibilities demonstrating that self-paced choice-to-choice temporal delays were not related to performance in either in the simple alternation task or in the delayed alternation task. This rules out passive decay models that are based solely on clock time. By contrast, imposing delays strongly affected performance even though the self-paced delays were often of a similar magnitude (up to a few minutes). Thus, our results favor an active interference account of the spatial working memory loss in the present T-maze task rather than elapsed time (Lewandowsky et al., 2009) whereby new information, originating internally or externally, pushes out old information.”

Figure 4d: There is no axis indicating the units for the x axis (counts of significant differences? T values? Inches? Percent of significant t-tests?) or where zero (or chance) would be given that this is stated to be the difference between left and right. Does the thick black line indicate zero? I thought that since differences within pig were computed then this could have positive or negative values but from the main text it seems that these are ether counts of the number of significant t-tests or the t values – so only positive. The specific measure should be clarified in the caption for 4d. The overall analysis is fairly clear in the main text.

> The bars are magnitudes representing the difference in distance between the average right and left turn location (x-axis) in each bin. A calibration bar has been added scaled to inches for simplicity. The stars indicate a significant difference in these magnitudes (one t-test per bin, df = 8, null = 0). The caption has been updated accordingly.

Related to the above: “Across bins, the number of significant t-tests per bin was as follows: Bin1 = 100%, Bin2 = 78%, Bin3 = 67%, Bin4 = 22%, Bin5 - B12 = 0% of pigs”. The percentages are listed here, not the number of significant t-tests.

> The t-test counts have been added.

Minor Points

Figure 1: I think that Ci and Cii could be combined more compactly in a single subplot. A lot of space is taken up by these simple schematic figures.

> These have been combined.

Figure 1: It would be nice to see a more close-up view of the pulley apparatus used to pull the guillotine doors and the feeding apparatus used to feed the pigs.

> Close-ups have been added.

Figure 4c: A scale bar would be useful (readers can try to align the image to Figure 4d, but it would be nice to have a scale bar in 4c).

> A calibration bar has been added.

Figure 4d: The “bin distance” label on the y axis is a little confusing. I think it would be clearer to label the y axis as just “Distance (inches)” and then keep the subheading that indicates that each bin was 12”.

> This was changed as suggested.

Minor: Technically, stating that “Female pigs were slightly more accurate (77.89 ± 2.22%, n = 6) than 110 male pigs (71.99 ± 2.07%, n = 3)” is not accurate given that there was no significant difference between groups. You could state that the mean performance was higher in the females and then state that the difference was not significant.

> This was corrected.

SWR should be defined.

> This was written out.

This heading was a little confusing: “No evidence that self-paced added delay durations impact performance”. Could be rewritten for clarity. ‘e.g., delays introduced by self-paced trials…’

> This was rewritten for clarity.

An exceedingly minor point that can be ignored: Seems like ‘overkill’ that video tracking + Matlab were used to control the apparatus when simple IR sensors with an Arduino or Raspberry Pi would be a simpler and less expensive solution for maze control. The camera of course is essential for trajectory tracking, just not necessary for experiment control.

> We agree. We have used IR sensors in the past with rats for a similar purpose. However, the pig maze was built with long term experimental goals that will benefit from detailed tracking (e.g., coupling behavior to ephys). But the point is well taken and CineLyzer software (Plexon) does not provide tracking with nearly the quality or reliability which we have been able to achieve with DeepLabCut software. Notably, DeepLabCut was not accessible when we first built the maze.

Page 7: only derivative measures were compared between pigs. Please be clear. I assume that by this it is meant that the difference (Euclidean in x) between the left and right was computed per pig and that is what was averaged across pigs?

> We updated this wording to be more specific.

This wording could be clarified “The durations were chosen to include multiple points double the last and followed basic behavioral guidelines for temporal behavioral tasks (Jayachandran & Allen, 2022).”

> This has been worded as follow: “In this phase delays of 5s, 60s, 120s, and 240s were imposed in the Start Area and presented randomly with replacement following recommended practices when designing temporal tasks (Jayachandran & Allen, 2022).”

No citation for pill bugs, fruit flies, and goldfish on page 3. Bees should be added- there is a considerable literature on bee decision making using the T or the Y configured as a T (central choice arm) version.

It is not entirely clear what all of the differences between Phase 1 and Phase 2 were beyond the 90sec interval and 120sec interval in Phase 2. Was randomization with replacement used in Phase 1 or some other approach?

> Phase 1 delays were also presented randomly with replacement. The difference is only in the durations and was used for only a couple sessions in order to acclimate the pig to the addition of delays.

Page 2: Typo with mouse and rat models having brains ~1/400th and ~1/500th the size of a human brain, respectively. The order must be reversed.

> Corrected.

Page 5: Minor but the statement, “pigs became more comfortable” verges on anthropomorphizing.

> This was changed to acclimated.

Page 9: Typo on line 8, “main tenants” should be “main tenets”

> This was removed in rewording the section.

Page 9 (top): Authors start with ‘First,’ and then jump right to ‘Third,’ with no ‘Second,’. This is probably just a typo.

> This was corrected.

For a given pig, was the side bias on trial 0 consistent across sessions? Just curious as to whether pigs have a strong initial-trial side bias that carries over from day to day.

> We looked at the first choices in pigs across all delay sessions. We found a tendency for pigs to be slightly biased to one side. Across pigs the mean choice bias (0 = left, 1 = right) was calculated as follows: 0.23, 0.47, 0.5, 0.62, 0.78, 0.75, 0.83, 0.83, 0.91. Sixty-three percent of first choices were repeats from the prior day. This pattern stands in stark contrast to the ~20% rate of errors (repeats) found during the delayed alternation sessions.

Reviewer: 2

The curious finding of behavioral tracking position/direction being predictive in the choice point but not start box (except for short delays) needs some further description. This result is also not mentioned in the Abstract). At first I thought that the pig could be adopting a strategy to point in the direction of its future choice in short delay trials, but then I realized that it couldn’t really do this because delays were not presented in blocks, but rather they were inter-mixed within a single session (Fig 1D). In explanation of this, authors write, “Thus, at short delays the ability to predict upcoming turns is an artifact of a continuous trajectory” but I do believe that the possibility of some kind of prospective choice strategy to pass the time (in this case short time) given previous trial success could be explored as a possibility. Does this tendency to predict performance based on automated tracking drop off steeply within the trial? More details can be provided. All I could find on this in the Results was “This shows that at short delays, there was some tendency toward bias position in the start area, but this tendency diminishes quickly as time goes on and is completely lost by D240.”

> The Abstract was updated to read, “Positional and head direction data could differentiate subsequent turns on short but not long delays.”

> The Discussion on this topic was expanded to read as follows:

> “Importantly, short delays do not provide time for the pig to settle into their preferred waiting location and the analysis is contaminated by the contiguous return paths from the last trial. In fact, the ability to discriminate subsequent turns on short delays provides a strong validation for our analytical approaches. That is, because pigs were highly successful, this performance biases return arm positions to the opposite side relative to their next successful choice. On short delays, the predictive value of positions and head directions is likely a consequence of this starting location bias, although it might also help the pig make the next choice. This behavioral bias is lost quickly as delays elapse and the initial advantage does persist into longer delays. Thus, at short delays the ability to predict upcoming turns might simply be an artifact of successful performance coupled to the maze design.”

Authors mention in discussion they measured error type, finding that ~7/8 errors were spontaneous, while 1/8 were perseverative (occurring after another error). This is interesting. Can authors provide more detail on individual animal performance on new errors vs. perseverative errors or some kind of trial +1 performance or win-stay, lose-switch strategy?

> Given the overall accuracy of the pigs there weren’t many error trials. The overwhelming proportion of spontaneous errors indicate a lose switch strategy. However, taken together with a dominant win-shift strategy on successful trials this seems to indicate pigs simply learned to alternate. The average percent perseverative errors for each pig were as follows: 3, 8, 13, 15, 15, 11, 12, 16 and 23%. This is now reported in the Results (Lines 178-180).

Minor

I would replace wording like ‘became more comfortable’ with ‘became acclimated’ to describe the effect of increasing familiarity and faster choice latencies.

> This has been updated as suggested.

Incomplete sentence: last sentence before the section “Neurocognitive assessments in pig models of neurological disease and mental health disorders” in the Discussion.

> This has been updated to read: “At the longer delays, the location in the start box does not predict future choices.”

Figure 2C caption, delete ‘time’ as latencies are mentioned.

> Deleted.

Figure 4F caption, start ‘arm’?

> This was corrected to “start area”.

Associate Editor: 1

Comments to the Author:

Overall, both reviewers expressed enthusiasm about these data, and both recommended more detail on methods. Reviewer 1 suggested additional analyses of head direction or 'heading' and Reviewer 2 suggested an additional analysis on subsequent trials/errors. These analyses could be very informative.

> These analyses were very informative and we believe the new data strengthen the paper.

**Revised Submission - OXFNSC-2023-007.R1**

**Decision letter - OXFNSC-2023-007.R1**

27-Sep-2023

Dear Dr. Allen,

It is a pleasure to accept your revised manuscript entitled "Testing Spatial Working Memory in Pigs using an Automated T-Maze" in its current form for publication in the Oxford Open Neuroscience. The comments of the reviewer(s) who reviewed your manuscript are included at the foot of this letter.

Please note that this journal operates with transparent peer review. This means that the full peer review history of your article will publish online alongside your article. This includes reviewer comments, editor decision letters, and your author responses.

Next steps

You will receive an email from no-reply@scipris.com within roughly one week. This is your invitation to sign up for an account with SciPris, Oxford University Press’ author portal hosted by Aptara. You will need to create an account if you do not already hold one. Please register or log into your account and follow the online instructions which will guide you through signing your licence and paying the APC. The email and the portal have clearly signposted support options if you need any help during this process.

Please note that SciPris is a completely different system from ScholarOne, so your credentials to submit your manuscript here will not work there. Once you’ve created a SciPris account, you will be able to use it whenever you publish with Oxford Open Neuroscience or any OUP journal. Please note that OUP will only ever request payment for applicable fees be made via SciPris or to an OUP bank account. If you ever have concerns about the legitimacy of a request, please do not hesitate to contact a customer services agent via the SciPris portal or directly via oupsupport@scipris.com.

Thank you for your fine contribution. On behalf of the Editors of the Oxford Open Neuroscience, we look forward to your continued contributions to the Journal.

Sincerely,

Dr. Alicia Izquierdo

Senior Editor, Oxford Open Neuroscience

aizquie@psych.ucla.edu

Associate Editor

Comments to the Author:

Author has addressed all the reviewer comments.
